# Estimating rice chlorophyll content and leaf nitrogen concentration with a digital still color camera under natural light

**DOI:** 10.1186/1746-4811-10-36

**Published:** 2014-11-06

**Authors:** Yuan Wang, Dejian Wang, Peihua Shi, Kenji Omasa

**Affiliations:** Institute of Soil Science, Chinese Academy of Sciences, 71 East Beijing Road, Nanjing, 210008 PR China; National Engineering and Technology Center for Information Agriculture, Nanjing Agricultural University, 1 Weigang Road, Nanjing, 210095 PR China; Graduate School of Agricultural and Life Sciences, The University of Tokyo, Yayoi 1-1-1, Bunkyo-ku, Tokyo, 113-8657 Japan

**Keywords:** Digital still color camera, Leaf color, Image processing technology, Natural light, Nitrogen, Rice

## Abstract

**Background:**

The color of crop leaves is closely correlated with nitrogen (N) status and can be quantified easily with a digital still color camera and image processing software. The establishment of the relationship between image color indices and N status under natural light is important for crop monitoring and N diagnosis in the field. In our study, a digital still color camera was used to take pictures of the canopies of 6 rice (*Oryza sativa* L.) cultivars with N treatments ranging from 0 to 315 kg N ha^-1^ in the field under sunny and overcast conditions in 2010 and 2011, respectively.

**Results:**

Significant correlations were observed between SPAD readings, leaf N concentration (LNC) and 13 image color indices calculated from digital camera images using three color models: RGB, widely used additive color model; HSV, a cylindrical-coordinate similar to the human perception of colors; and the L^*^a^*^b^*^ system of the International Commission on Illumination. Among these color indices, the index b^*^, which represents the visual perception of yellow-blue chroma, has the closest linear relationship with SPAD reading and LNC. However, the relationships between LNC and color indices were affected by the developmental phase. Linear regression models were used to predict LNC and SPAD from color indices and phasic development. After that, the models were validated with independent data. Generally, acceptable performance and prediction were found between the color index b^*^, SPAD reading and LNC with different cultivars and sampling dates under different natural light conditions.

**Conclusions:**

Our study showed that digital color image analysis could be a simple method of assessing rice N status under natural light conditions for different cultivars and different developmental stages.

**Electronic supplementary material:**

The online version of this article (doi:10.1186/1746-4811-10-36) contains supplementary material, which is available to authorized users.

## Background

Nitrogen (N) is one of the most important nutrients essential for the growth of crops, and is a major component of chlorophyll and protein which are closely associated with leaf color, crop growth status and yield [1]. Insufficient N supply leads to smaller leaves, lower chlorophyll content and less biomass production, and consequently, reduced grain yield and quality [2, 3]. Conversely, excessive N application can lead to the environmental problems of water and atmospheric pollution [4]. Hence, measuring crop N status timely is critical for increasing N use efficiency and environmental quality [5, 6].

The spectral reflectance of crop leaf or canopy is known to be correlated with N status [7, 8]. The instruments for measuring spectral reflectance are the chlorophyll meter [9–11], multi-spectral sensor [12], hyper-spectral sensor [7, 8] and commercial digital camera [13–15], are used in precision agriculture for growth monitoring, nitrogen diagnosis and site-specific crop management. The SPAD meter (SPAD-502, Minolta Camera Co., Osaka, Japan), measures leaf chlorophyll content nondestructively, has potential for improving N use efficiency without affecting grain yield in real-time nitrogen management (RTNM) experiments [9, 11]. A disadvantage of the SPAD-502 for assessing crop N status is its small sampling area (6 mm^2^). In addition, the measurements are subject to operator bias so a large number of repetitions are needed to obtain reliable results [16, 17], and SPAD meter experiences difficulties in distinguishing chlorophyll levels when crops are near or above the optimum N supply [10, 18]. In contrast, satellite or airborne-mounted hyperspectral sensors can obtain spectral information in a larger sampling area and record more spectral bands [7, 8, 12]. The high cost of images, infrequent satellite overpasses and risk of images being obscured by clouds limit the application of these platforms for commercial use [19]. The proximal sensors GreenSeeker® (NTech Industries Inc., USA) and Yara N-sensor (Yara International ASA, Germany) which measure red and near infra-red (NIR) reflectance, overcome some of the limitations of satellite or airborne sensors [20], but their accuracy is influenced by background soil interference [8].

Alternatively, images from digital still color cameras, which record spectral information of visible bands, have a low cost but very high image resolution (consumer cameras in 2014 record up to 40 megapixels per image). At a sensor height of about 1 meter above the canopy, high-spatial-resolution images separate crops from background soil or other interferences, which is important for accurate diagnosis of N status when the vegetation fraction was low [13, 21]. Moreover, images from digital still cameras contain a large amount of information about the crop structure and leaf color, such as leaf orientation, plant height, biomass accumulation and leaf senescence [22–24], and these parameters are easy to obtain [13, 25] with existing software, such as MatLab® (MathWorks Inc.), the free-ware package ImageJ [26]. Previous studies showed that canopy cover estimated from the images was not only highly correlated with leaf area index (LAI), aboveground biomass and N accumulation [13, 27], but was also stable in varying environmental conditions [28, 29]. In addition to canopy cover, color digital images provide spectral information in the visible bands which are closely related to the leaf N concentration (LNC) and SPAD readings [17]. Hunt *et al.* [30] found that the triangular greenness index (TGI), which was derived from red, green and blue bands of a digital still color camera, was sensitive to leaf chlorophyll content of a whole canopy.

The color-related indices from digital still cameras can diagnose crop N status [17, 30, 31]. Previous studies on the analysis of leaf color were mostly undertaken in controlled light conditions [17, 32–34]. This approach could reduce the impact of light on the image color, and easily obtain a reliable relationship between N status and leaf color indices. However, the results derived from the controlled light cannot be completely applied to natural light because of the variable light conditions [15, 35, 36]. Besides, there are still many uncertainties in the use of digital still cameras for N diagnosis under natural light conditions, and further validation is necessary to ensure the application in the field.

In this study, experiments with different N application rates were carried out in the field under sunny and overcast conditions (1) to analyze the relationship between chlorophyll content, LNC and canopy color related indices in different cultivars and stage of phasic development, (2) to establish the possible models for the diagnosis of crop N status using image color indices, and (3) to validate the applicability of the models under different natural light conditions.

## Results and discussion

### Correlation of color related indices and crop nitrogen status

Correlations between two rice N parameters (leaf N concentration and SPAD reading) and 13 image-color related indices (Eq. 1–10) were calculated with individual and pooled cultivars in 2011. Similar results were obtained from the three rice cultivars, i.e., Liangyoupeijiu, Nanjing45 and Nanjing46. Therefore, only correlation coefficients from the Liangyoupeijiu dataset (n = 72) and the pooled dataset (n = 240) were displayed in Table 1. SPAD readings showed significant correlations with each color index except g in both datasets. Among these color indices, L^*^, b^*^, R, G, B, r and INT were negatively correlated with SPAD readings, while the other indices were positively correlated with SPAD readings. Most of the color indices were closely correlated with LNC, however, the magnitude and direction of the correlation coefficients were not consistent with those between color indices and SPAD readings. Overall, the indices derived from the CIE L^*^a^*^b^*^ color model had relatively higher correlation coefficients with SPAD readings and LNC. In this color model, the index b^*^ represents the visual perception of yellow-blue chroma which is similar with the leaf color variation, and it has been used in many other studies for image color analysis [37, 38]. Therefore, we select index b^*^ as a representative for further analysis.

**Table 1:** **Correlation coefficients between SPAD readings, leaf nitrogen concentration (LNC, g kg** ^**-1**^ **) and image color related indices (digital number from three color models: RGB, HSV and CIE** ***L*** ^*^ ***a*** ^*^ ***b*** ^*^ **)**

	SPAD	LNC	***L*** ^*^	***a*** ^*^	***b*** ^*^	***b*** ^*^ ***/a*** ^*^	H	R	G	B	r	g	b	INT	VI _Green_
SPAD	1.00	0.21	-0.67^**^	0.78^**^	-0.90^**^	0.76^**^	0.83^**^	-0.68^**^	-0.67^**^	-0.48^**^	-0.76^**^	0.01	0.41^**^	-0.63^**^	0.66^**^
LNC	0.32^**^	1.00	0.48^**^	-0.26^*^	-0.06	0.35^**^	0.13	0.47^**^	0.48^**^	0.63^**^	-0.37^**^	-0.68^**^	0.65^**^	0.52^**^	-0.28^*^
*L* ^*^	-0.62^**^	0.35^**^	1.00	-0.83^**^	0.73^**^	-0.43^**^	-0.63^**^	1.00^**^	1.00^**^	0.96^**^	0.38^**^	-0.64^**^	0.21	1.00^**^	-0.90^**^
*a* ^*^	0.61^**^	-0.04	-0.70^**^	1.00	-0.86^**^	0.50^**^	0.69^**^	-0.82^**^	-0.84^**^	-0.69^**^	-0.52^**^	0.18	0.17	-0.80^**^	0.62^**^
*b* ^*^	-0.73^**^	-0.35^**^	0.54^**^	-0.72^**^	1.00	-0.86^**^	-0.95^**^	0.74^**^	0.73^**^	0.51^**^	0.87^**^	0.04	-0.51^**^	0.68^**^	-0.73^**^
*b* ^*^ */a* ^*^	0.60^**^	0.48^**^	-0.29^**^	0.36^**^	-0.91^**^	1.00	0.96^**^	-0.45^**^	-0.43^**^	-0.18	-0.99^**^	-0.26*	0.72^**^	-0.37^**^	0.63^**^
H	0.61^**^	0.40^**^	-0.39^**^	0.42^**^	-0.91^**^	0.97^**^	1.00	-0.64^**^	-0.63^**^	-0.40^**^	-0.95^**^	-0.09	0.58^**^	-0.58^**^	0.75^**^
R	-0.63^**^	0.33^**^	1.00^**^	-0.68^**^	0.56^**^	-0.34^**^	-0.44^**^	1.00	1.00^**^	0.96^**^	0.39^**^	-0.64^**^	0.21	1.00^**^	-0.92^**^
G	-0.62^**^	0.35^**^	1.00^**^	-0.71^**^	0.54^**^	-0.29^**^	-0.39^**^	1.00^**^	1.00	0.96^**^	0.38^**^	-0.64^**^	0.21	1.00^**^	-0.90^**^
B	-0.37^**^	0.58^**^	0.91^**^	-0.47^**^	0.15^*^	0.09	-0.03	0.90^**^	0.91^**^	1.00	0.12	-0.82^**^	0.48^**^	0.98^**^	-0.83^**^
r	-0.56^**^	-0.54^**^	0.19^**^	-0.35^**^	0.89^**^	-0.99^**^	-0.95^**^	0.24^**^	0.19^**^	-0.20^**^	1.00	0.36^**^	-0.79^**^	0.31^**^	-0.56^**^
g	0.05	-0.66^**^	-0.62^**^	0.03	0.31^**^	-0.44^**^	-0.34^**^	-0.60^**^	-0.61^**^	-0.87^**^	0.56^**^	1.00	-0.86^**^	-0.69^**^	0.58^**^
b	0.27^**^	0.69^**^	0.27^**^	0.17^**^	-0.66^**^	0.79^**^	0.71^**^	0.23^**^	0.26^**^	0.63^**^	-0.87^**^	-0.90^**^	1.00	0.29^*^	-0.08
INT	-0.56^**^	0.42^**^	0.99^**^	-0.64^**^	0.45^**^	-0.20^**^	-0.31^**^	0.99^**^	0.99^**^	0.95^**^	0.10	-0.70^**^	0.37^**^	1.00	-0.89^**^
VI_Green_	0.67^**^	-0.10	-0.85^**^	0.42^**^	-0.65^**^	0.62^**^	0.68^**^	-0.88^**^	-0.85^**^	-0.68^**^	-0.51^**^	0.43^**^	0.02	-0.83^**^	1.00

Numbers in the upper diagonal were calculated with the Liangyoupeijiu (n = 72) dataset and numbers in the lower diagonal were calculated with the combined data of Liangyoupeijiu, Nanjing45 and Nanjing46 in 2011 (n = 240).

^**^indicate the significance at *P* < 0.01, ^*^indicate the significance at *P* < 0.05.

### Relationships between SPAD, LNC and the color index b^*^

**Figure 1:**
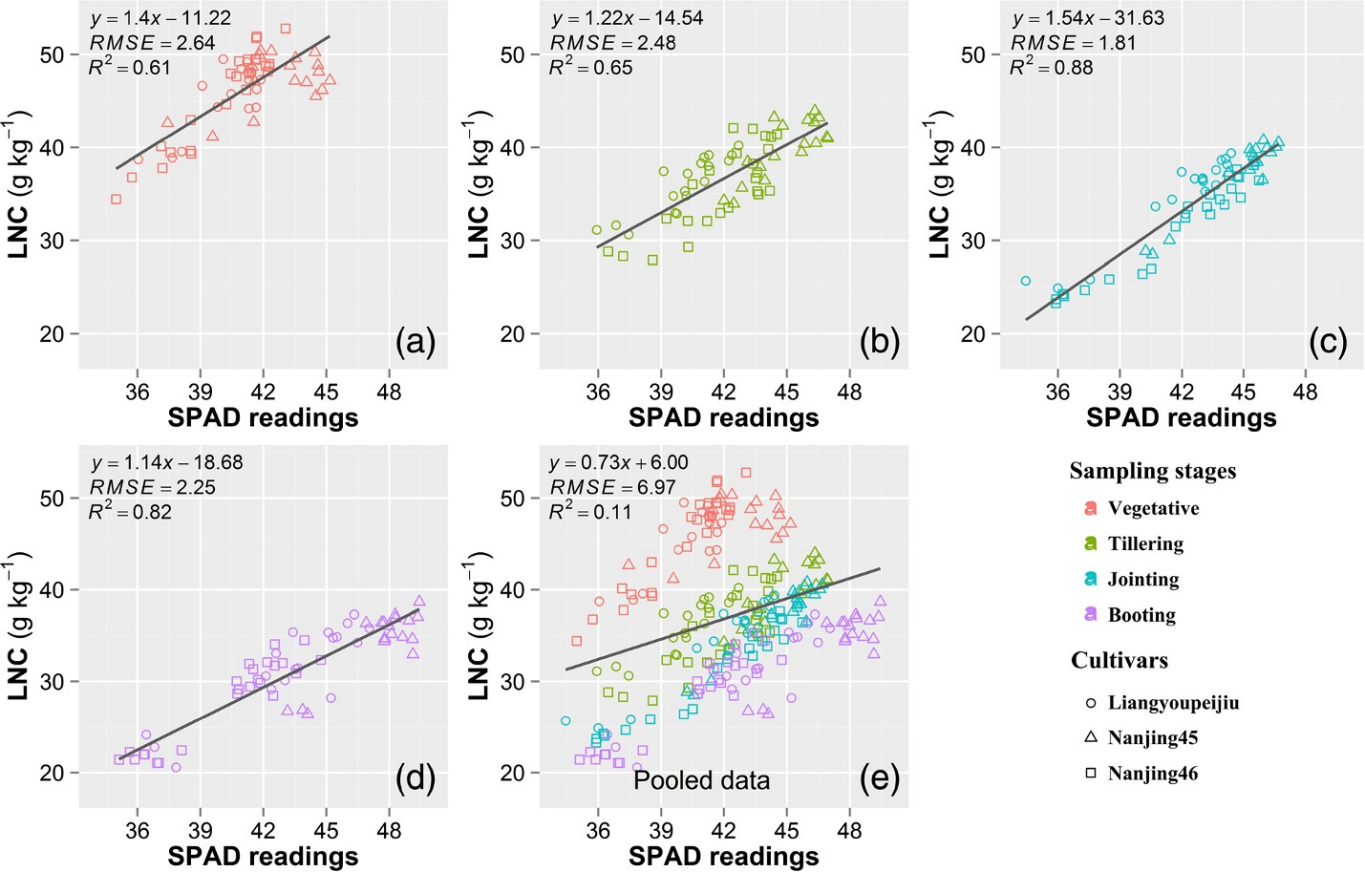
**Relationships between leaf nitrogen concentration (LNC, g kg** ^**-1**^ **) and SPAD readings in vegetative (a), tillering (b), jointing (c), booting (d) stages, and the pooled data of the four stages (e) in 2011.**

Regression analyses were performed between SPAD readings, LNC and the color index b^*^ using the 2011 dataset. Positive linear relationships were observed between LNC and SPAD readings, with the same trends in different sampling dates and cultivars (Figure 1). The determination coefficient (R^2^) in different sampling dates varied from 0.61 to 0.88 along with the root mean square error (RMSE) from 1.81 to 2.64 g kg^-1^. The LNC decreased with the rice development, while the maximum SPAD values increased with rice growth. Smaller RMSE was obtained in jointing and booting stages than in vegetative and tillering stages. Similarly, Xue et al. [39] reported that the ratio index of NIR/green (R_810_/R_560_) reached the best accuracy with LNC at jointing stage. When data were pooled across the sampling dates, there was no significant trend observed between SPAD readings and LNC (Figure 1e).

**Figure 2:**
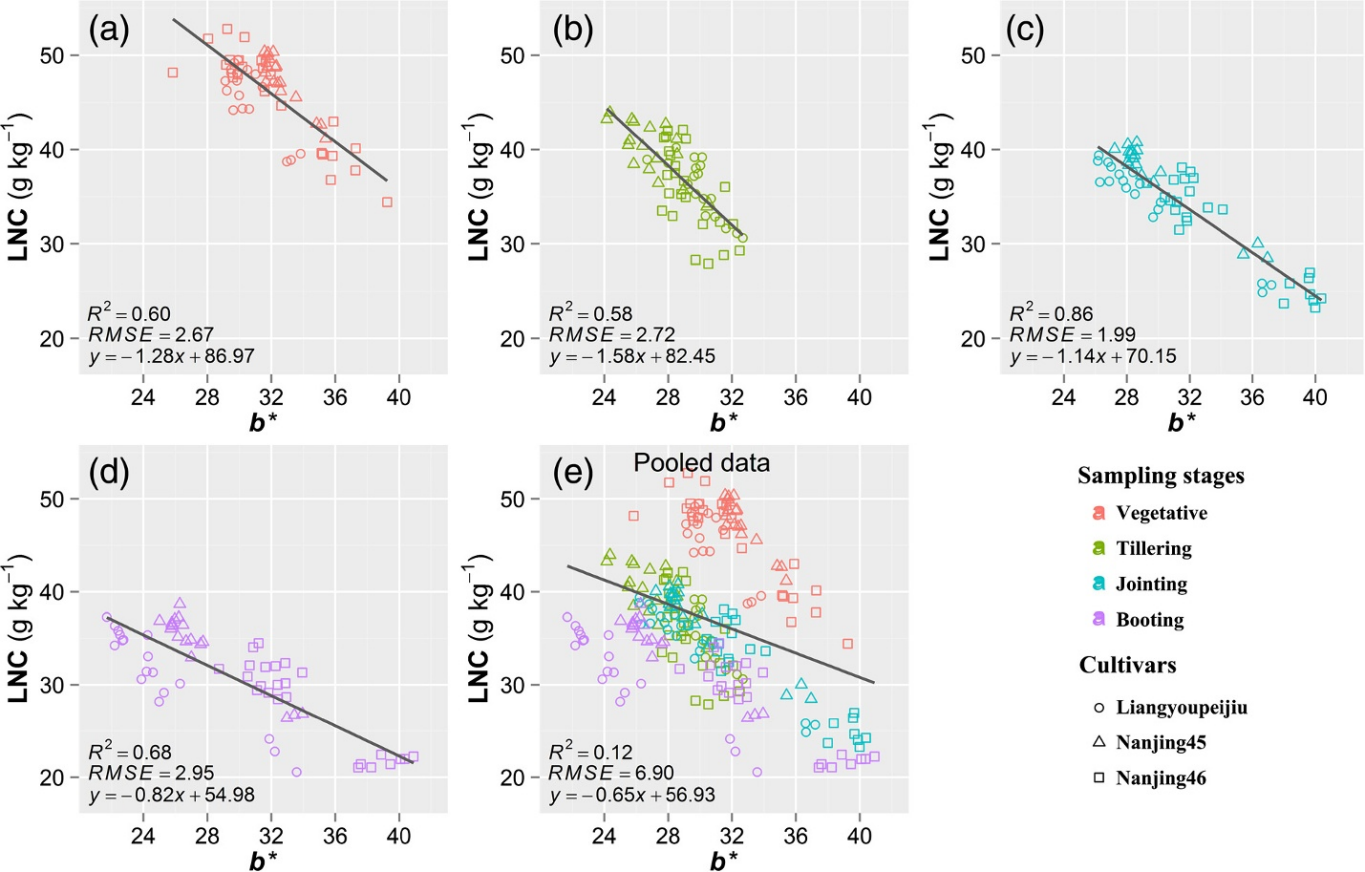
**Relationships between leaf nitrogen concentration (LNC, g kg** ^**-1**^ **) and image color index** ***b*** ^*^ **in vegetative (a), tillering (b), jointing (c), booting (d) stages, and the pooled data of the four stages (e) in 2011.**

**Figure 3:**
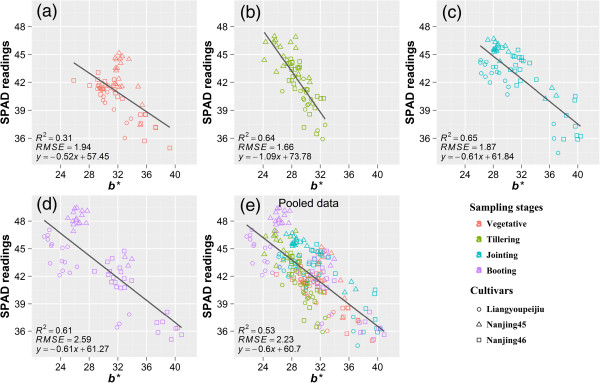
**Relationships between SPAD readings and image color index** ***b*** ^*^ **in vegetative (a), tillering (b), jointing (c), booting (d) stages, and the pooled data of the four stages (e) in 2011.**

Significant negative linear relationships were seen between LNC and color index b^*^, with R^2^ ranging from 0.58 to 0.86 in the four developmental stages (Figure 2). The R^2^ between b^*^ and LNC at booting stage (Figure 2d) was less than that between SPAD readings and LNC. Same as the relationship between SPAD readings and LNC, no significant trend was observed between b^*^ and LNC when data pooled across the sampling dates (Figure 2e). In addition, there were negative linear relationships between b^*^ and SPAD readings (Figure 3). The R^2^ in the vegetative stage was lower than that in the other stages. There were obvious differences among cultivars for the relationship between b^*^ and SPAD readings, especially the Liangyoupeijiu in jointing and booting stages (Figure 3c and d) and the Nanjing45 in vegetative stage (Figure 3a). In this case, the regression analysis was carried out with individual cultivars (Table 2). Overall, higher R^2^ were observed from individual cultivars and sampling dates than that from the pooled dataset (Table 2). However, the intercepts and slopes of the linear relationship varied with rice cultivars, these differences might be partly caused by the different plant type among cultivars, with a loose shape and large mean leaf angle in hybrid indica rice (Liangyoupeijiu) [40, 41] while tight shape and small mean leaf angle in japonica rice (Nanjing45, Nanjing46). The different plant type lead to different distribution of reflectance [42, 43] and finally caused different image-derived indices.

**Table 2:** **Statistics of the linear regression analysis between color index** ***b*** ^*^ **and SPAD readings in different development stages and cultivars in 2011**

		Vegetative	Tillering	Jointing	Booting	All stages
Liangyoupeijiu	R^2^	0.74^**^	0.62^**^	0.92^**^	0.86^**^	0.80^**^
	RMSE^a^	0.87	1.18	0.86	1.23	1.24
Nanjing45	R^2^	0.42^**^	0.28^**^	0.90^**^	0.79^**^	0.60^**^
	RMSE	1.61	1.41	0.64	0.91	1.60
Nanjing46	R^2^	0.83^**^	0.52^**^	0.82^**^	0.83^**^	0.59^**^
	RMSE	0.94	1.62	1.47	1.26	1.85
All cultivars	R^2^	0.31^**^	0.64^**^	0.65^**^	0.61^**^	0.53^**^
	RMSE	1.94	1.66	1.87	2.59	2.23

Dependent variable: SPAD.

^**^indicate the significance at *P* < 0.01.

^a^Root mean square error.

In our experiments, image acquisition was carried out in the field under natural light near solar noon which was the period with the most stable illumination at the top of the atmosphere. This makes sure that the light intensity is not changing too much during image acquisition in a single day. The results from Table 2 indicated that reliable estimates of N status could be obtained from images taken under natural light. Considering the stability of N diagnosis in different locations and sampling dates, individual sampling dates could not meet the needs of crop monitoring and N diagnosis in various environmental conditions. Therefore, regression analysis was carried out with the pooled data of sampling dates. There were large differences of light intensity among different sampling dates even under overcast days (Table 3, PAR ranging from 145 to 692 *μ*mol m^-2^ s^-1^ in 2011).

**Table 3:** **Rice cultivars, sampling dates (indicated as days after transplanting, DAT), photosynthetic active radiation (PAR,** ***μ*** **mol m** ^**-2**^ **s** ^**-1**^ **) and the number of samples in the two experiments**

Experiment	Year	Cultivar	Vegetative	Tillering	Jointing	Booting	Number of samples
I	2010	Wuyunjing24	25^a^ (937)^b^	36 (1536)	50 (1369)	64 (1532)	18
	2011	Nanjing46	18 (145)	35 (692)	55 (203)	75 (296)	24
II	2010	Nanjing44	22 (1215)	35 (1058)	49 (1759)	63 (1477)	18
		Yangjing48	22 (1449)	35 (1058)	49 (1759)	63 (1477)	18
	2011	Nanjing45	16 (427)	29 (654)	51^c^ (589)	57 (289)	18
		Liangyoupeijiu	16 (427)	29 (654)	51 (589)	65 (621)	18

^a^represents sampling dates (the days after transplanting, DAT).

^b^represents the average PAR during the period of image acquisition.

^c^Because of the continuous sunny days in the jointing stage for Nanjing45, sampling dates was delayed about a week.

Interestingly, regression analysis did not show any evidence that the relationship between SPAD and color index b^*^ was affected by the varying light intensity (Figure 3e). This might be attributed to the auto exposure controlled by the digital camera which adjusted the exposure time to make compensation for the amount of light reaching the image sensor. However, there were no significant trends between LNC and SPAD readings, or between LNC and color index b^*^ using data pooled across different sampling dates (Figures 1e and 2e). Previous studies revealed that, for rice and corn, the relationship between LNC and SPAD readings could be improved simply by dividing the readings with specific leaf weight (SLW = dry leaf weight/leaf area) of the sampled leaves or introducing SLW as a second independent variable in the multiple regression [44–46]. The reason is that SPAD readings vary with leaf thickness which can be different in cultivars, developmental stages and environmental conditions [46, 47], while the LNC has a relatively consistent value. The uncertain relationship between LNC and color index b^*^ (Figure 2e) may also be caused by the difference of leaf thickness, because the color index b^*^ and SPAD readings both reveal the spectral information of leaves, and their relationship keeps consistent with the pooled data of different sampling dates (Table 2 and Figure 3e). Nowadays, destructive sampling or hyperspectral-reflectance [48] is required for the measurement of SLW, however, including this defeats the purpose of using a cheap and simple digital camera.

### Model calibration and validation

**Figure 4:**
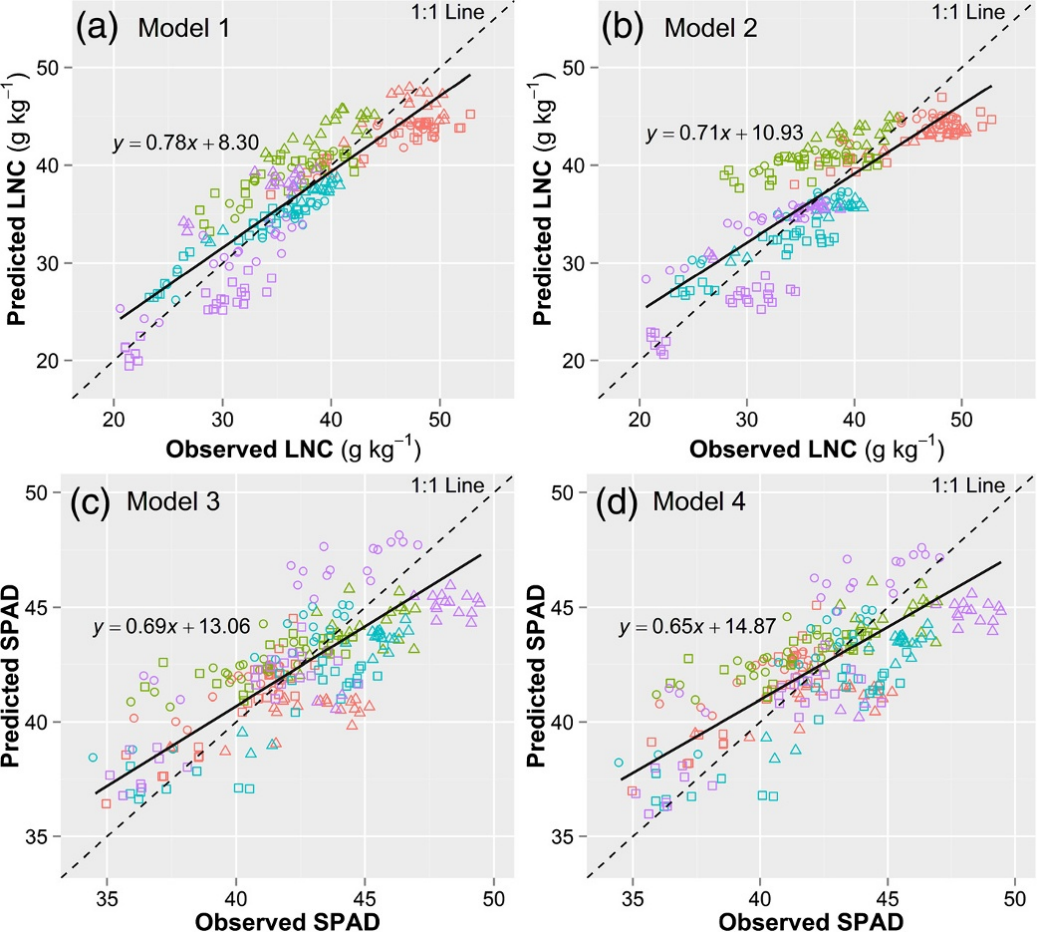
**Calibration of the four models in Table** 4 **for the estimation of leaf nitrogen concentration (LNC, g kg** ^**-1**^ **) and SPAD.** Model 1 **(a)**, *LNC* = α*SPAD* + β*DAT* + γ, model 2 **(b)**, *LNC* = α*b* ^*^ + β*DAT* + γ, model 3 **(c)**, *SPAD* = α*b* ^*^ + β*DAT* + γ, model 4 **(d)**, *SPAD* = α*b* ^*^ + γ. Different colors denote different developmental stages (red: vegetative, green: tillering, blue: jointing, purple: booting). Different symbols denote different cultivars used for model calibration (○ Liangyoupeijiu, ∆ Nanjing45, □ Nanjing46).

Since developmental stages in rice affected the response of N parameters to color indices, multiple linear regression analysis was carried out with the consideration of days after transplanting (DAT) to estimate LNC and SPAD (Table 4). The multiple linear models highly improved the R^2^ of SPAD readings and b^*^ with LNC (Table 4, Figure 4a and b). The relationships between SPAD readings and b^*^ were less affected by the process of development for all the cultivars (Table 2 and Figure 3e). Therefore, the consideration of DAT in the multiple linear regression did not improve the R^2^ effectively (Figure 4c). The slope of the regression lines in Figure 4 was all less than 1, which indicated that predicted LNC or SPAD were generally smaller at the high value area and bigger at the low value area, than the observed ones. It was noteworthy that most of the predicted LNC in the vegetative stage (red symbols) were underestimated in Figure 4a and b. This is mainly because that the plants at this stage has higher concentration of N but lower concentration of chlorophyll. In this case, the lower concentration of chlorophyll would cause an underestimation of the LNC in the vegetative stage.

**Table 4:** **Statistics of the calibration and validation results for estimating leaf nitrogen concentration (LNC, g kg** ^**-1**^ **) and SPAD with color index** ***b*** ^*^ **, SPAD and days after transplanting (DAT)**

Models	α	β	γ	RMSE ^a^	R ^2^	NMB ^b^
*Calibration*						
Model 1 *LNC* = α*SPAD* + β*DAT* + γ	1.02 ± 0.07	-0.31 ± 0.01	7.01 ± 2.95	3.07 g kg^-1^	0.78	
Model 2 *LNC* = α*b* ^*^ + β*DAT* + γ	-0.67 ± 0.06	-0.29 ± 0.01	69.27 ± 2.10	3.36 g kg^-1^	0.71	
Model 3 *SPAD* = α*b* ^*^ + β*DAT* + γ	-0.60 ± 0.04	0.024 ± 0.007	59.66 ± 1.14	1.62	0.55	
Model 4 *SPAD* = α*b* ^*^ + γ	-0.60 ± 0.04		60.70 ± 1.12	1.62	0.53	
*Validation*						
Model 1				2.43 g kg^-1^	0.75	1.19%
Model 2				2.59 g kg^-1^	0.62	-1.32%
Model 3				2.01	0.46	-1.94%
Model 4				1.89	0.47	-2.00%

The dataset in 2011 was used for model calibration and the dataset in 2010 used for model validation.

^a^Root mean square error.

^b^Normalized mean bias.

**Figure 5:**
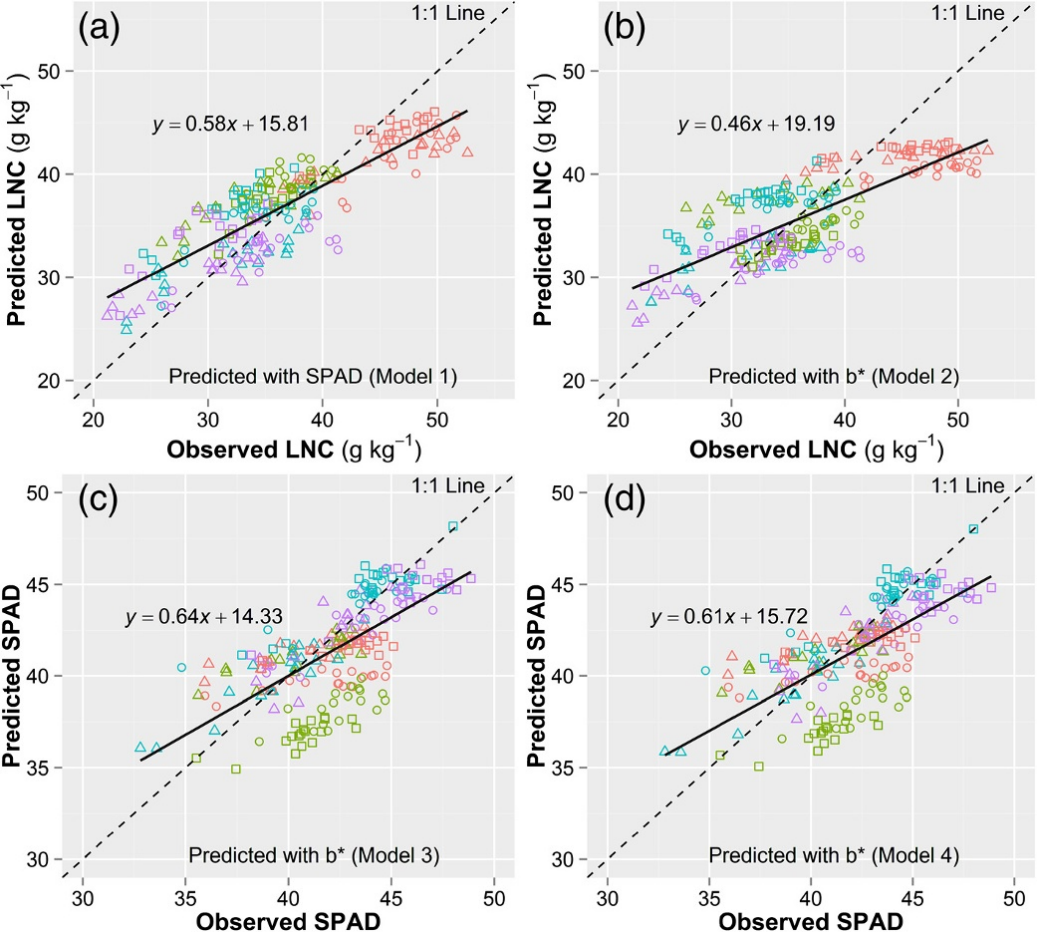
**Validation of the four models using the fitted parameters in Table** 4 **for the estimation of leaf nitrogen concentration (LNC, g kg** ^**-1**^ **) and SPAD.** Model 1 **(a)**, *LNC* = α*SPAD* + β*DAT* + γ, model 2 **(b)**, *LNC* = α*b* ^*^ + β*DAT* + γ, model 3 **(c)**, *SPAD* = α*b* ^*^ + β*DAT* + γ, model 4 **(d)**, *SPAD* = α*b* ^*^ + γ. Different colors denote different developmental stages (red: vegetative, green: tillering, blue: jointing, purple: booting). Different symbols denote different cultivars used for model validation (○ Nanjing44, ∆ Wuyunjing24, □ Yangjing48).

Validations were performed on the four models in Table 4 with all data obtained in 2010 under sunny days. The images used in model calibration and validation were taken under different weather conditions, the objective of this combination was to evaluate whether the model was robust under different light conditions. In general, good performances on the predicted models were observed for the estimation of LNC and SPAD (Figure 5). Model 1 showed the best performance on the prediction of LNC with a normalized mean bias of 1.19% (Table 4 and Figure 5a). Model 2, 3 and 4 showed relatively lower R^2^ and smaller negative bias. The model for the prediction of LNC with color index b^*^ (Figure 5b) was not severely affected by the different light conditions in 2010 and 2011, compared to the prediction of LNC with SPAD (Figure 5a). As with the calibration results, most of the data in the vegetative stage were below the 1:1 line in models 1 and 2. In addition, the data in tillering stage deviated from the 1:1 line in models 3 and 4, which resulted in the low R^2^ (Figure 5). The similar R^2^ and RMSE in Figure 5c and d indicated that the relationship between SPAD and color index b^*^ was not affected by the developmental stage.

In our study, the light conditions during image acquisition were different between 2010 and 2011. Image acquisition in 2011 was under overcast days with low but stable diffused light. In contrast, images were taken under sunny days in 2010 with strong and variable (PAR ranging from 937 to 1759 *μ*mol m^-2^ s^-1^) light (Table 3). We can remove the effect of illumination change on images when the change is over the whole image by normalizing the image [49] or using a calibration panel [36]. However, there were many other differences caused by the different light conditions. The strong illumination in 2010 caused many white spots and shadows which affected the image color (Additional file 1: Figure S1) while this phenomenon was hardly seen in the images taken in 2011. With the rice growth, the light status within rice canopy (transmittance, reflectance, absorption) becoming more and more complex, the change of illumination will cause different degrees of influence on canopies that in different height (Additional file 2: Figure S2). In this case, it is difficult to calibrate the image color accurately. Sakamoto *et al.* [15] calibrated image indices with the introduction of exposure value (EV) and obtained reliable camera retrieved vegetation indices (VIs). However, this method cannot apply to our study for the calibration of canopy color because of the different influence in one image.

Besides the light conditions, the prediction of crop N status with leaf color may be affected by many other environmental factors, such as developmental stage, diseases and drought stress [50]. These factors may be detectable from high-resolution canopy images [22, 23]. Color indices analysis associated with these factors and other image characteristics (e.g. canopy cover, plant shape, leaf texture or even soil status), will provide more reliable results to N diagnosis. Further studies will be devoted to the exploration of image characteristics, leaf color correction and the calibration of the established model with physiological parameters (e.g. SLW) in the evaluation of N status under different environmental conditions.

## Conclusions

Image color indices calculated from RGB, HSV and L^*^a^*^b^*^ color models have significant correlations with SPAD readings and leaf N concentration (LNC) of rice leaves. Among these color indices, the index b^*^, which represents the visual perception of yellow-blue chroma, had the highest correlation coefficients with SPAD readings and LNC. Regression analysis showed significant linear relationships between index b^*^ and N parameters. However, the relationship between LNC and SPAD reading, LNC and index b^*^ were affected by the rice developmental stage. This is mainly caused by the leaf thickness which can be different in cultivars, developmental stages and environmental conditions. In this case, linear regression models were established between color index b^*^, LNC and SPAD readings by considering the developmental process in rice. The multiple linear models improved the R^2^ of SPAD readings and b^*^ with LNC, yet most of the predicted LNC in the vegetative stage were underestimated because of the inconsistent relationship between chlorophyll and N concentration. Validations on the models showed good performance and acceptable predicted precision with different cultivars and sampling dates under different natural light conditions. These results indicated that digital color image analysis could be a simple method for assessing rice N status under natural light conditions.

## Materials and methods

### General information of the experimental site

The experiment was laid out at Changshu Agricultural Ecology Experiment Station, Changshu, Jiangsu, China (31°33′N, 120°42′E). Located in the humid subtropical climate zone, the station receives average annual solar radiation of 4930 MJ m^-2^, sunshine of 1800 hours, precipitation of 1200 mm and cumulative temperature above 10°C of 4933 degree-days (°C·d). The soil type for the field experimental site is a gleyed paddy soil of the Taihu Lake region, which contains total nitrogen (N) of 1.79 g kg^-1^, total phosphorus (P) of 0.93 g kg^-1^, total potassium (K) of 18.7 g kg^-1^, organic matter of 30.8 g kg^-1^, alkali-extractable N of 123 mg kg^-1^, Olsen-P of 13.1 mg kg^-1^, plant available K of 121 mg kg^-1^ and pH of 7.4 (soil: water, 1:2) in the 0–15 cm soil layer.

### Experimental design

Two independent experiments with different N fertilization gradients were implemented in our study. Experiment I was a long-term site-specific rice-wheat rotation experiment that started in 1997. The trial comprised six fertilizer treatments represented as CK, N0, N1, N2, N3, and N4 for N application of 0, 0, 180, 225, 270 and 315 kg N ha^-1^ in rice season, respectively. Each treatment had four replicates which were arranged in a randomized block design. The data used in this paper were from the period May to November in 2010 and 2011 with cultivars Wuyunjing24 and Nanjing46, respectively. Experiment II was carried out in paddy fields with a rice-wheat rotation in 2010 and 2011. Six N application rates with three replicates were designed in this trial, which were represented as N0, N1, N2, N3, N4 and N5 with N application of 0, 120, 180, 240, 270 and 300 kg N ha^-1^, respectively. The cultivars were Nanjing44 and Yangjing48 in 2010, and Nanjing45 and Liangyoupeijiu in 2011. For both experiments, the N was split into three applications, 40% as basal, 20% at tillering and 40% at booting. In addition, each plot received 90 kg K ha^-1^ and 20 kg P ha^-1^ except the CK treatment in experiment I. The applied K was split into 50% as basal and 50% at booting, and all the P was applied as basal fertilizer. Other crop managements were same as the local traditional practices.

### Sample collection and digital image acquisition

For measuring rice growth and nutrition parameters, the above-ground part of rice plant was sampled about every two weeks after transplanting until the booting stage. A total of 4 sets of samples were collected in 2010 and 2011 (Table 3). The plant samples were separated into leaves and stems (including sheaths), and dried at 105°C for half an hour and then at 70°C until constant weight. After that, the samples were weighed for dry weight and analyzed for leaf N concentration (LNC) by the Kjeldahl method [51]. Along with the plant sampling, a chlorophyll meter (SPAD-502, Minolta Camera Co., Osaka, Japan) was used to obtain SPAD values on the four youngest fully expanded leaves. Each blade was measured at three points: on the upper, middle and lower thirds on either side of the midrib. Then, average SPAD readings were calculated for each plot.

On the same day or following day of plant sampling, images of the rice canopy were captured using a digital still color camera (EOS 50D, Canon Inc.) with a resolution of 15 mega pixels. The camera was mounted on a tripod at the nadir position with a constant height of 1 m above the top of the rice canopy. Aperture priority mode was selected, and the camera was set at aperture of f/5.6, ISO of 100, white balance of 4,900 K, auto exposure and auto-focus with the flash turned off. In 2010, the pictures were taken at local time 12:00 – 13:00 in sunny days, while in 2011, the pictures were taken at the same time period but on overcast days. In the days of picture taken (July and August), the deviation between local time and solar noon was within 4 minutes. All the pictures from the experiments were stored in CR2 (Canon raw image file) format. The photosynthetic active radiation (PAR) and illuminance were recorded by a portable light meter (GLZ-C, Top Instrument Co., Zhejiang, P. R. China) during the period of image acquisition. Average PAR was calculated with each set of pictures, and observed 937–1759 *μ*mol photons m^-2^ s^-1^ and 145–692 *μ*mol photons m^-2^ s^-1^ in 2010 and 2011, respectively (Table 3).

### Image segmentation and color indices calculation

A raw image file contains minimally processed data from the image sensor of a digital camera. This file saves settings of white balance, color saturation, contrast and sharpness in it, but defers the processing. Therefore, all the modification made on a raw image file is non-destructive.

The canopy images in CR2 format were adjusted for white balance using the 18% gray card (R-27, Kodak) pictures which were taken simultaneously with the canopy images. Then, lens distortion correction was applied, and exposure was set to +1 for all images. After that, images were saved as joint photographic experts group (JPEG) files for further processing. All the procedures above were processed with Adobe Camera Raw (Adobe Systems Inc.).

Since the images contained the rice canopy and some non-canopy elements, such as soil, water and plant residues, images were segmented into canopy portion and non-canopy portion. A computer program was developed based on the G-R thresholding method [25, 29] using MatLab® (MathWorks Inc.) to extract the canopy portion of the image. The G-R thresholding method was proposed according to the difference of reflectance spectrum between green vegetation and non-canopy elements in the visible band. There is a reflection peak for green vegetation in the green band, whereas no apparent change for soil or water albedo in the whole visible band. Therefore, the value of green channel minus that of red channel expands the difference between canopy and non-canopy portion.

After the image segmentation, 13 color indices derived from 3 color models were calculated. RGB model is the most common color model for the representation of digital images. A color in the RGB model is described by indicating how much of each of the red, green, and blue is included. The color is expressed as an RGB triplet (R, G, B), with the representation for black of (0, 0, 0) and for the brightest representable white of (255, 255, 255) in an 8-bit image [52]. R, G and B are the mean values of the red, green and blue channels, and r, g and b are the normalized RGB values, respectively. Intensity (INT) is the average of R, G and B. VI_Green_ is a widely used vegetation index [13]. These indices were calculated as follows [27, 29]:
12345

In addition, the CIE L^*^a^*^b^*^ and HSV color spaces were also tested in this study. The L^*^ coordinate in CIE L^*^a^*^b^*^ [53] closely matches human perception of lightness, a^*^ and b^*^ dimensions represent the visual perception of red-green and yellow-blue chroma, respectively. Both a^*^ and b^*^ are independent with image lightness (L^*^), and take on both negative and positive values (+a^*^ reds, - a^*^ greens, + b^*^ yellows, - b^*^ blues). The three coordinates of L^*^a^*^b^*^ are computed from the tristimulus values X, Y and Z as following equations [32, 54]:
6789

where *X*_*n*_, *Y*_*n*_ and *Z*_*n*_ describe a specified white object-color stimulus.

The HSV color space is represented as a cylindrical-coordinate in which the angle around the central vertical axis corresponds to hue (H). The calculation of H was listed below [27, 29]:
10

Pearson correlation and regression analyses were used to detect the relationship between color indices and crop N status. The significance of linear regressions was evaluated using Student’s *t*-test at 95% confidence levels. Significance of ANOVAs was evaluated with the least significant difference test (LSD) at 0.05 probability level. Data analysis and figure production were done using the R v3.0.3 software [55].

Correlation analysis, linear regression analysis and model establishment between color indices and crop N status were based on the data in 2011, and the data in 2010 were used for model validation.

## Electronic supplementary material

Additional file 1: Figure S1: Examples of the “white spots”, which are over-exposed areas where the reflected light came into the camera directly. (PDF 691 KB)

Additional file 2: Figure S2: Canopy images of Nanjing46 in different developmental stages (a, vegetative; b, tillering; c, jointing; d, booting). (PDF 1 MB)

## Abbreviations

N: Nitrogen
LNC: Leaf nitrogen concentration
H: Hue from HSV color space
R: G, B: Digital number for the red, green and blue channel of an RGB image
r: g, b: Normalized RGB values
INT: The average of R, G and B.
