# Supplementary material for: Estimating rice chlorophyll content and leaf nitrogen concentration with a digital still color camera under natural light
Source: Plant Methods. 2014 Nov 6;10:36. doi: 10.1186/1746-4811-10-36 (PMC4236477; doi:10.1186/1746-4811-10-36)
Supplement: Supplementary file 1 — Additional file 1: Figure S1: Examples of the “white spots”, which are over-exposed areas where the reflected light came into the camera directly. (PDF 691 KB) [file 13007_2014_304_MOESM1_ESM.pdf]

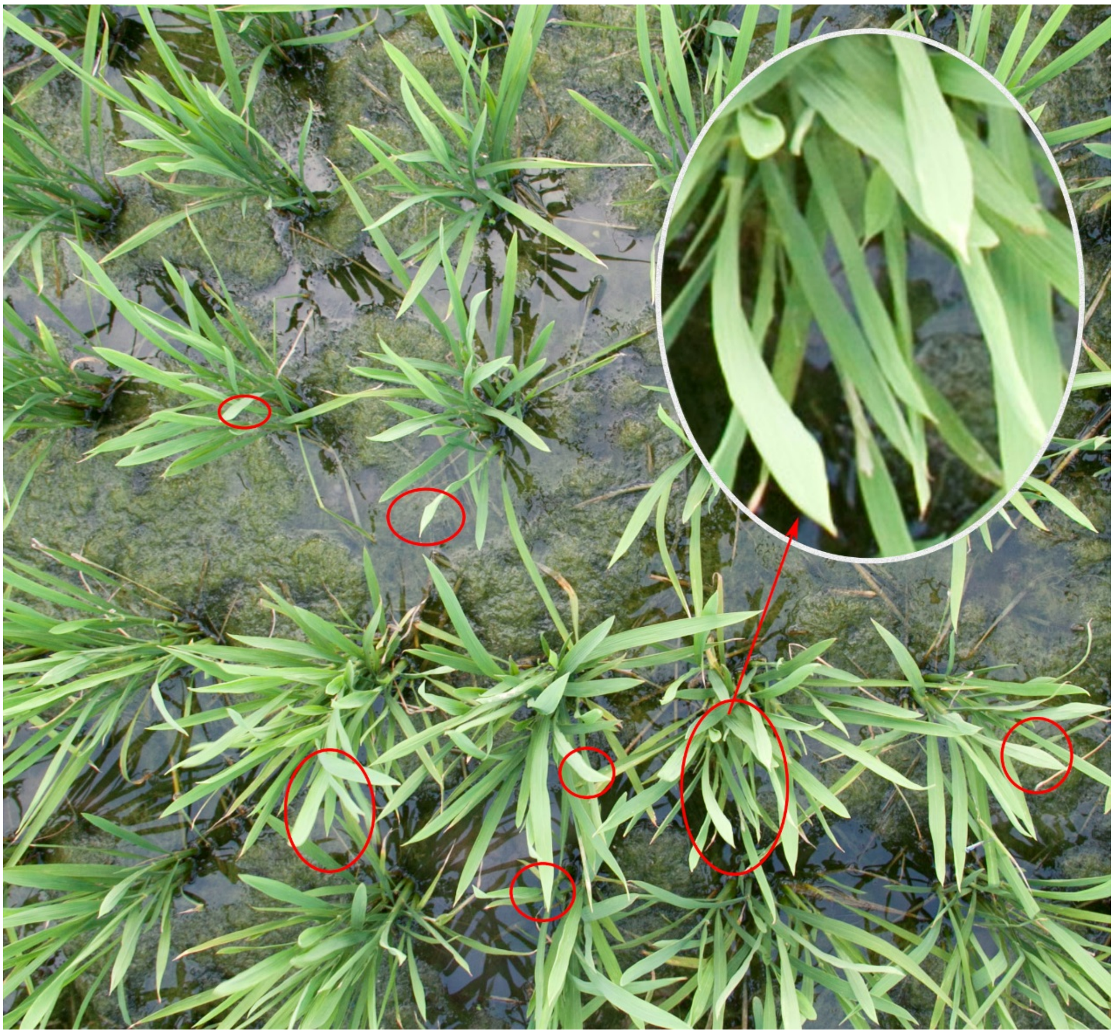

Figure S1. Examples of the "white spots", which are over-exposed areas where the reflected light came into the camera directly.
