# Supplementary material for: Estimating rice chlorophyll content and leaf nitrogen concentration with a digital still color camera under natural light
Source: Plant Methods. 2014 Nov 6;10:36. doi: 10.1186/1746-4811-10-36 (PMC4236477; doi:10.1186/1746-4811-10-36)
Supplement: Supplementary file 2 — Additional file 2: Figure S2: Canopy images of Nanjing46 in different developmental stages (a, vegetative; b, tillering; c, jointing; d, booting). (PDF 1 MB) [file 13007_2014_304_MOESM2_ESM.pdf]

a

b

c

d

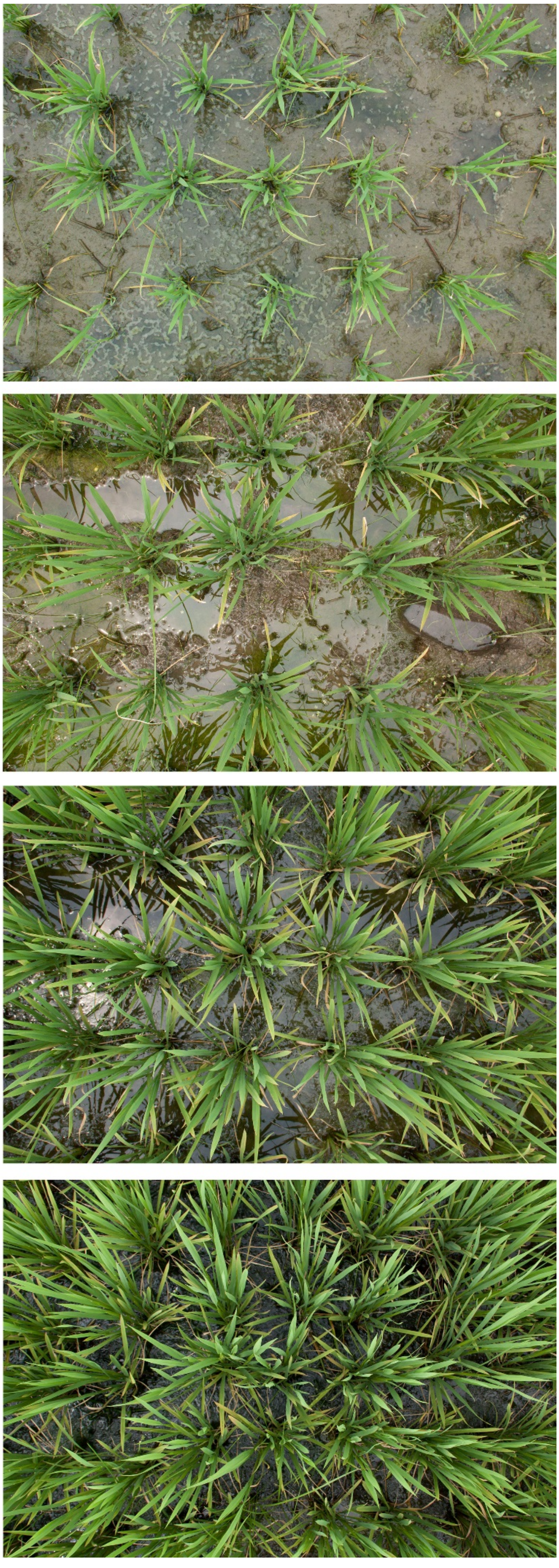

Figure S2. Canopy images of Nanjing46 in different developmental stages (a, vegetative; b, tillering; c, jointing; d, booting).
